# Phenotypic high-content screening identifies plant-derived extracts targeting microbial growth, biofilms and swarming activity

**DOI:** 10.3389/fmicb.2026.1898345

**Published:** 2026-09-04

**Authors:** Julia Kirchsteiger, Verena Preinfalk, Gerald Klanert, Elisabeth Kohberger, Clemens Schwarzinger, Julian Weghuber, Bettina Schwarzinger, Alice König

**Affiliations:** 1 FFoQSI GmbH – Austrian Competence Centre for Feed and Food Quality, Safety and Innovation, Wels, Austria; 2 Center of Excellence Food Technology and Nutrition, University of Applied Sciences Upper Austria, Wels, Austria; 3 Institute for Chemical Technology of Organic Materials, Johannes Kepler University, Linz, Austria

**Keywords:** antimicrobial plant extracts, biofilm, broth microdilution, disk diffusion, high-content screening, pathogens, swarming

## Abstract

The rise of antimicrobial resistance highlights the urgent need for new antibacterial agents, particularly from natural sources. In this study, 200 aqueous plant extracts were screened for antimicrobial activity against six clinically relevant pathogens. The test organisms included: *Escherichia coli* ATCC35401, *Pseudomonas aeruginosa* DSM19882, *Salmonella enterica* subsp. *enterica* ser. Typhimurium ATCC14028, *Staphylococcus aureus* NCCB32030, *Streptococcus mutans* DSM20523 and *Enterococcus faecalis* DSM2570. Three probiotic lactobacilli (*Lacticaseibacillus paracasei*, *Lactiplantibacillus plantarum* and *Lacticaseibacillus rhamnosus*) were used to differentiate pathogen-specific activity from broad-spectrum inhibition. Antimicrobial activities were initially assessed using the agar disk diffusion method, followed by broth microdilution to determine minimum inhibitory concentrations (MICs) and minimum bactericidal concentrations (MBCs) for active plant extracts. Additionally, the effects on biofilm formation by *E. coli*, *S.* Typhimurium, *S. aureus* and *S. mutans*, and on swarming motility of *P. aeruginosa* and *S.* Typhimurium, were evaluated. 21 extracts inhibited at least one pathogen in the disk diffusion assay. Defined MIC values were detected for 19 extracts, while only 5 demonstrated bactericidal activity. In addition to the known antibacterial plants *Allium sativum* and *Allium ursinum*, previously unreported leaf extracts from *Castanea sativa*, *Ribes × nidigrolaria* and *Rubus laciniatus* showed promising activity against the tested pathogens. These extracts not only inhibited bacterial growth but also reduced biofilm formation and swarming motility of some pathogenic species at subinhibitory concentrations. Notably, no inhibition of *Lactobacillus* growth was detected in the disk diffusion assay. Overall, our findings highlight the potential of aqueous plant extracts as sources of antimicrobial and anti-virulence activities and demonstrate the importance of pathogen-specific effects in the discovery of plant-derived antimicrobials.

## 1 Introduction

Despite considerable efforts to improve production and hygiene standards as well as to promote consumer awareness, food spoilage and food-associated pathogenic microorganisms continue to cause substantial economic losses and unacceptable health consequences worldwide (Bouarab Chibane et al., 2019). Traditionally, food spoilage and the associated pathogens have frequently been controlled using conventional antibiotics. However, their excessive use has resulted in antibiotic residues in food products and has significantly contributed to the emergence of antimicrobial resistance (AMR; Ahmed et al., 2024; Mostafa et al., 2018). As microorganisms can rapidly adapt to changing environmental conditions and transfer resistance genes through mechanisms such as horizontal gene transfer, bacterial infections are becoming increasingly difficult to treat. Consequently, AMR is considered one of the greatest global health threats of the 21^*st*^ century. Without effective countermeasures, antimicrobial resistance could result in approximately 10 million deaths annually by 2050 (Ahmed et al., 2024). In response to this growing challenge, the World Health Organization (WHO) has established a priority list of antibiotic-resistant bacteria to promote and guide the development of novel antimicrobial agents (SeyedAlinaghi et al., 2025).

In this context, plant extracts are increasingly being regarded as promising alternatives. They provide natural antimicrobial compounds, are generally considered safe for consumption, and are readily biodegradable (Mostafa et al., 2018). The biological activity of plants is primarily attributed to secondary plant metabolites, also referred to as phytochemicals. In particular, plant-derived compounds such as alkaloids, phenolic compounds, and terpenoids are considered promising sources of novel antimicrobial agents and may either act directly as antibacterial substances or synergistically enhance the activity of existing antibiotics by interfering with bacterial virulence mechanisms (Woo et al., 2023). Traditionally used medicinal plants such as garlic, ginger, green tea and turmeric have demonstrated antimicrobial properties and may serve as valuable lead structures for future drug development (Vladkova et al., 2024). In addition, novel antimicrobial compounds continue to be discovered. For example, one study investigated extremophilic plants from southern Tunisia and demonstrated that these desert plants may represent a potential source of new antimicrobial molecules and contribute to the development of innovative strategies against antibiotic-resistant pathogens (Ben Brahim et al., 2026). Collectively, these findings highlight the potential of natural compounds as antimicrobial agents.

Among the most important bacterial pathogens associated with foodborne diseases are Gram-negative bacteria such as *Salmonella* ssp., *Escherichia coli* (*E. coli*), and *Pseudomonas aeruginosa* (*P. aeruginosa*), as well as Gram-positive bacteria such as *Staphylococcus aureus* (*S. aureus*). Many of these pathogens are capable of producing toxins, forming spores, or developing biofilms, thereby increasing their resistance to heat and cleaning agents (Phan et al., 2025). Accordingly, the present study focused on clinically and food-relevant bacterial pathogens including *E. coli, P. aeruginosa, Salmonella enterica* subsp. *enterica* ser. Typhimurium (*S.* Typhimurium), *Enterococcus faecalis* (*E. faecalis*), *S. aureus*, and *Streptococcus mutans* (*S. mutans*).

*Escherichia coli*, *S.* Typhimurium, and *P. aeruginosa* are Gram-negative bacteria associated with intestinal, foodborne, and nosocomial infections. These pathogens exhibit diverse virulence mechanisms, including host cell invasion, biofilm formation, and increased antibiotic resistance, which contribute to their persistence and pathogenicity (Devlin et al., 2022; Doing et al., 2023; Madhavan and Sakellaris, 2015). Due to its multidrug resistance and adaptive capabilities, *P. aeruginosa* has additionally been classified by the WHO as a critical priority pathogen requiring the development of novel antimicrobial agents (Mane et al., 2020). *E. faecalis* and *S. aureus* are Gram-positive bacteria and important opportunistic pathogens frequently associated with nosocomial infections and increasing antimicrobial resistance (Ahmad-Mansour et al., 2021; Lee et al., 2003; Loghmani et al., 2024; Ullah et al., 2024). In addition, both species exhibit pronounced biofilm-forming abilities that contribute to their persistence and pathogenicity (Ahmad-Mansour et al., 2021; Ullah et al., 2024). *S. mutans*, in contrast, is a Gram-positive oral pathogen involved in dental plaque biofilm formation and enamel demineralization leading to dental caries (Lemos et al., 2019; Lin et al., 2021).

The pronounced virulence properties and adaptive survival mechanisms exhibited by many pathogenic bacteria highlight the need for alternative therapeutic approaches targeting bacterial pathogenicity rather than bacterial viability alone. Accordingly, anti-virulence strategies that interfere with pathogenic traits such as quorum sensing, biofilm formation, or motility are receiving growing attention, as they may reduce pathogenicity without exerting strong selective pressure on microorganisms (Vale et al., 2016). Biofilm formation represents a widespread microbial survival strategy that enables protection against adverse environmental conditions. In pathogenic bacteria, biofilm formation substantially contributes to antibiotic tolerance and elevated pathogenicity (Shamim et al., 2023). In recent years, biofilm dispersal has gained increasing scientific attention, as understanding this process is important for explaining infection dynamics, developing anti-biofilm strategies, and improving knowledge of bacterial ecology and antibiotic resistance (Sahu et al., 2025). Another important virulence factor is swarming motility, which refers to the rapid and coordinated multicellular movement of bacteria across moist surfaces driven by rotating flagella and is widespread among bacterial species (Irazoki et al., 2016). Swarming enables the spatial expansion of bacterial populations and can result in distinct colony morphologies, including dendritic and fractal-like patterns (Kasallis et al., 2023). Furthermore, swarming has been associated in many flagellated bacteria with host surface colonization, increased virulence factor expression, and enhanced antibiotic resistance. This adaptive, non-genetic resistance has been described in species such as *Salmonella enterica*, *E. coli*, and *P. aeruginosa*, where the resistant phenotype disappears once the bacteria are cultivated under non-swarming conditions (Irazoki et al., 2017).

In addition to conventional antimicrobial assays, such as agar disk diffusion and broth microdilution (Balouiri et al., 2016), alternative approaches including anti-swarming and anti-biofilm assays are gaining increasing importance (Vale et al., 2016). Therefore, the aim of this study was to perform a high-content screen of 200 plant extracts prepared at different extraction temperatures to evaluate their antimicrobial activity against clinically and food-relevant bacterial pathogens. The extracts were obtained from well-known plant species selected based on their regional availability and their potential applicability, as the respective plants are generally considered edible and non-toxic. Extracts showing inhibitory activity were further evaluated regarding their minimum inhibitory and bactericidal concentrations, their effects on swarming motility and biofilm formation, as well as their selectivity toward beneficial lactobacilli in the disk diffusion assay.

## 2 Materials and methods

### 2.1 Preparation of plant extracts

Plant material was mainly collected in the region of Upper Austria, gently dried either at 50 °C overnight (herbs and spices) or in a dehydrator for 18–48 h (vegetables, fruits and other), and ground using a commercial coffee grinder. Plant extracts were prepared under cold and hot conditions as previously described (Blank-Landeshammer et al., 2024; Neuhauser et al., 2024; Steinbauer et al., 2024). Briefly, 5 g of dried plant powder were mixed with 30 mL of either water at 22 °C (“cold extracts”) or water at 70 °C (“hot extracts”). For cold extraction, the samples were placed in the ultrasonic bath for 30 min, followed by overhead shaking for 2 h. For hot extraction, the samples were thoroughly mixed in the overhead shaker for 10 min. The extraction performed at 22 °C is hereafter designated as extraction method 1, whereas the extraction conducted at 70 °C is designated as extraction method 2. All extracts were vortexed and centrifuged at 4,149 × *g* for 10 min. Two further extraction steps were carried out before the combined supernatant was filtered to a final volume of 50 mL. One batch per extract was prepared. The final extract had a mass-solvent ratio of 1:10 and was used as 100% extract stock. Aliquots of the extracts were stored at −80 °C until further use. Prior to further testing, all extracts were sterilized by filtration through 0.22 μm membrane filters.

The pH value was measured at room temperature using a calibrated pH meter, while the dry matter content was determined at 110 °C on 2 g of extract using a moisture analyzer (MA 160 Moisture Analyzer, Sartorius, Göttingen, Germany). The dry extract concentration of the active extracts was determined gravimetrically. A defined volume of extract was dried at 110 °C in a drying oven until constant weight was achieved. The resulting dry residue was weighed, and the dry extract concentration was expressed as mg dry extract per mL.

Total phenolic content (TPC) was determined using Folin-Ciocalteu according to Blank-Landeshammer et al. (2024), with gallic acid as the calibration standard. Absorbance was measured at 750 nm, and results were expressed as mg gallic acid equivalents (GAE) per g dry weight. Total antioxidant activity was determined by the ferric reducing antioxidant power (FRAP) assay according to a previously published method (Heckmann et al., 2024), using ( ± )-6-hydroxy-2,5,7,8-tetramethylchroman-2-carboxylic acid (TROLOX) as the calibration standard. Absorbance was measured at 593 nm, and results were expressed as TROLOX equivalents (TE) per g dry weight.

### 2.2 Evaluation of antimicrobial activity

The antibacterial activity of plant extracts against the following six clinically and food-relevant pathogens was assessed: *Escherichia coli* ser. O78:H11 ATCC35401 (American Type Culture Collection, ATCC, Manassas, VA, USA), *Pseudomonas aeruginosa* PA14 DSM19882 (German Collection of Microorganisms and Cell Cultures GmbH, DSMZ, Braunschweig, Germany), *Salmonella enterica* subsp. *enterica* ser. Typhimurium ATCC14028 (ATCC), *Staphylococcus aureus* NCCB32030 (Westerdijk Fungal Biodiversity Institute, WI-KNAW, Utrecht, Netherlands), *Streptococcus mutans* DSM20523 (DSMZ) and *Enterococcus faecalis* DSM2570 (DSMZ). The three probiotic lactobacilli *Lacticaseibacillus paracasei* DSM20312 (DSMZ), *Lactiplantibacillus plantarum* DSM20205 (DSMZ) and *Lacticaseibacillus rhamnosus* NCTC10302 (National Collection of Type Cultures, NCTC, Salisbury, UK) were used as control bacteria to distinguish extracts with pathogen-specific antimicrobial activity from those with a broader inhibition spectrum.

#### 2.2.1 Agar disk diffusion method

The antibacterial activity of 200 aqueous plant extracts was evaluated according to the EUCAST disk diffusion method for antimicrobial susceptibility testing (version 9.0). The bacteria were streaked on Mueller-Hinton (MH) agar (Carl Roth GmbH, Karlsruhe, Germany) using a sterile inoculation loop on the previous day and incubated at 35 °C for 20 ± 4 h. Bacterial suspensions were prepared by transferring 4–8 morphological similar colonies into 0.85% NaCl solution and adjusted to a 0.5 McFarland turbidity standard (1–2 × 10^8^ CFU/mL). The turbidity was measured using a densitometer (McFarland densitometer DEN1, Grant-bio, Grant Instruments Ltd., England) and a deviation of ± 0.1 was tolerated. The freshly prepared bacterial suspensions were evenly distributed on MH agar plates with sterile cotton swabs using a plate rotator (petriturn-E, schuett-biotec GmbH, Germany). Blank filter paper disks (Oxoid Ltd., England) were placed on the surface of the inoculated agar using a multidisk dispenser (6 cartridges, 90 mm, Oxoid) and 12.5 μL of undiluted plant extracts were added to each disk. All plates were tested in triplicates and included the antibiotic gentamycin (Gen) (ROTI^®^ Antibiotic Disks, 10 μg Gentamycin, Carl Roth) as positive control and distilled water as negative control (in the center of the plate). After incubation at 35 °C for 18 ± 2 h, the antibacterial activity of the extracts was evaluated by determining whether a zone of inhibition around the disks was visible. Extracts that caused an inhibition zone against at least one of the pathogens were considered a positive hit.

#### 2.2.2 Determination of MIC and MBC

The minimum inhibitory concentration (MIC) and minimum bactericidal concentration (MBC) of the positive hits were determined against the sensitive pathogens by the broth microdilution method according to the Clinical and Laboratory Standards Institute (CLSI) guidelines with minor modifications. Briefly, the inoculum of the bacteria was prepared from 24 h old culture plates, adjusted to a 0.5 McFarland turbidity standard and further diluted (1:250,000) in Mueller-Hinton-Bouillon 2 (MH2, Sigma-Aldrich, St. Louis, MO, USA). Serial dilutions of plant extracts (50%–0.05%) were prepared in a 96-well plate using a two-fold dilution series. Finally, 50 μL of fresh bacterial suspension were added to each well, except to blank wells that contained extract but no bacteria. Growth control wells did not contain extracts but only bacteria. Gentamycin sulfate (≥590 I.U./mg, Carl Roth) diluted in sterile water was used as positive control in a concentration range of 50 μg/mL to 0.05 μg/mL. The final volume in each well was 100 μL. After incubation at 35 °C for 16 ± 2 h, MICs were determined as the lowest concentration of extract that displayed no visible growth. The turbidity (optical density at 600 nm, OD_600_) was recorded with the Tecan Infinite M200 plate reader (Tecan Group, Männedorf, Switzerland). To obtain the MBC, samples from wells with no visible growth were subcultured onto MH agar plates. The lowest concentration of extract that resulted in no visible growth of colonies after 24 h of incubation at 35 °C was defined as the MBC. Extracts were tested in triplicates per plate, and each experiment was independently repeated three times.

### 2.3 Measurement of anti-biofilm activity

Fresh overnight cultures (ONCs) were prepared in Luria-Bertani medium (LB, Carl Roth) and incubated for 18–20 h at 30 °C (*E. coli*) or 37 °C (*S.* Typhimurium, *S. aureus, S. mutans*). Bacterial cultures of *E. coli, S.* Typhimurium and *S. aureus* were adjusted to an OD_600_ of 0.02 in modified basal medium 2 (BM2), prepared as described previously (Haney et al., 2018). The final glucose concentration in the medium was 0.4% for *E. coli* and *S.* Typhimurium as well as 0.8% for *S. aureus*. Bacterial cultures of *S. mutans* were adjusted to an OD_600_ of 0.02 in tryptic soy broth (TSB, Fisher Scientific GmbH, Vienna, Austria) with 1% sucrose. Subsequently, 50 μL of culture were added to 50 μL of medium containing either solvent control, the respective positive control or plant extracts in a transparent flat-bottom 96-well plate, leading to a final OD_600_ of 0.01. Phloretin (Extrasynthese, Lyon, France), quercetin or xylitol (both Sigma-Aldrich) were used as positive control. In order to verify that the positive controls were specific to the different strains, they were tested in advance for their biofilm-inhibiting effects. Plant extract concentrations were selected based on preliminary toxicity tests in the same media and did not affect bacterial growth (growth ≥ 80% of control). For *E. coli*, wells were pre-coated with 200 μL of sterile-filtered 0.1% bovine serum albumin (BSA, Sigma-Aldrich) at 37 °C for 20 min prior to inoculation.

To allow bacterial growth and biofilm formation, plates were incubated under static conditions at 30 °C for 24 h (*S.* Typhimurium) or 37 °C for 48 h (*E. coli*, *S. aureus*, *S. mutans*). Bacterial growth was quantitated by measuring OD_600_. The medium containing planktonic cells was discarded and the adhered biomass was washed three times with 250 μL sterile phosphate-buffered saline (PBS, PAN-Biotech, Aidenbach, Germany). Biofilms were stained with 120 μL of 0.1% crystal violet for 15–20 min, followed by three additional PBS washing steps. Plates were air-dried for 1 h and the retained stain was solubilized with 120 μL of 30% acetic acid. A total of 100 μL of the resulting homogeneous solution was transferred to a new 96-well plate for measurement of OD_585_. Wells containing sterile medium, positive controls or extracts without bacteria were used as blanks and subtracted from sample readings.

Biofilm formation was calculated as ratio of OD_585_ (crystal violet) to OD_600_ (bacterial growth) to normalize biofilm biomass to culture growth and presented relative to the untreated control (biofilm formed in the absence of extracts). The relative reduction in biofilm biomass was expressed as percentage inhibition. Extracts were tested in quadruplicates per plate, and each experiment was independently repeated three times.

### 2.4 Evaluation of swarming motility

The swarming motility was assessed using agar plate assays with species-specific media. The preparation of the subcultures was adapted from the method described by Partridge and Harshey (2023), whereas incubation parameters such as temperature and incubation time, as well as the used agar and drying times of the plates were optimized experimentally in this study. Overnight cultures were prepared by inoculating 5 μL of bacterial glycerol stock into 5 mL of LB medium and incubating at 35 °C under shaking at 200 rpm. For subculturing, overnight cultures were diluted 1:100 in fresh LB medium (50 μL culture in 4.95 mL LB) and grown at 35 °C with shaking at 200 rpm until reaching mid-exponential phase (OD_600_ ≈ 0.6). Swarm agar plates were prepared freshly before each experiment by pouring 20 mL of medium per plate. For *S.* Typhimurium, Eiken agar was used, whereas *P. aeruginosa* was cultivated on M9 agar plates. The tested plant extracts were incorporated directly into the molten agar at the respective concentrations before pouring the plates. After solidification, plates were dried for approximately 30 min prior to inoculation. After the drying time, a volume of 6 μL of the subculture was carefully spotted onto the center of the agar surface without damaging the agar and the plates were incubated at 35 °C for 17 ± 1 h. Untreated cultures served as growth controls, and sodium salicylate (15 mM for *S.* Typhimurium and 35 mM for *P. aeruginosa*) served as positive control.

The swarming area on the plates was quantitated using the open-source software ImageJ/Fiji (Schindelin et al., 2012). Swarming was expressed as the percentage of the total agar plate area covered by bacterial growth. Raw images were used for quantitative analysis. However, to improve visual presentation, representative images were adjusted to achieve equal brightness, contrast, and intensity to avoid misinterpretations.

### 2.5 Cytotoxicity assay in mammalian cells

The cytotoxicity of selected plant extracts was assessed in human Caco-2 epithelial cells (DSMZ) using a resazurin-based *in vitro* toxicology assay (Sigma-Aldrich) according to the manufacturer’s instructions. Briefly, 196,430 cells/cm^2^ were seeded into black 96-well plates with a clear bottom in Eagle Minimum Essential Medium (EMEM, +Earle’s balanced salts, +2 mM L-glutamine, +non-essential amino acid, +1 mM sodium pyruvate, + 1.5 g/L sodium bicarbonate) supplemented with 10% fetal bovine serum (FBS) and 1% penicillin-streptomycin (P/S) (all from PAN-Biotech), and incubated overnight at 37 °C in a humidified atmosphere with 5% CO_2_. Then, cells were washed with PBS and exposed to extract solutions diluted 1:100 in cell culture medium for 30 min at 37 °C. After treatment, the extract solutions were removed and cells were incubated with 50 μM resazurin in cell culture medium for 90 min at 37 °C. The amount of resofurin formed was quantified with a POLARstar Omega microplate reader (BMG Labtech) in fluorescence mode at 544 nm excitation and 590 nm emission wavelength. Results were normalized to the average of the control. A cell viability over 80% was considered as non-cytotoxic.

### 2.6 Chemical-analytical characterization of plant extracts

For liquid chromatography – tandem mass spectrometry (LC-MS/MS) analyses the extracts were filtered using syringe filters (RC, 0.2 μm). Samples were analyzed by reversed-phase chromatography using a Surveyor HPLC (Thermo Fisher Scientific, Waltham, MA, USA) equipped with an Accucore C18 column (150 mm × 3.0 mm i.d., 2.6 μm particle size; Thermo Fisher Scientific). The column temperature was set to 40 °C and the injection volume was 1 μL. The analytes were separated by gradient elution with mobile phase A containing 0.1% formic acid (FA) in water and mobile phase B containing 0.1% FA in acetonitrile at a flowrate of 0.5 mL/min. The elution gradient starting conditions were 95% A and 5% B. After 5 min of isocratic phase, the proportion of B was increased to 20% at 8 min and to 40% at 12 min, followed by 60% at 15 min and 80% at 17 min for 3 min. B was reduced to 5% at 20 min until 25 min. High-resolution mass spectra were obtained using an LTQ Orbitrap Velos (Thermo Fisher Scientific) with an APCI source operated in positive and negative ionization mode. The resolution was set to 30 000 and di-isooctylphthalate (m/z = 391.2843) was used as an internal standard for mass calibration. Spectra were collected from 100 to 2 000 m/z and MS^2^ spectra were automatically recorded from the most intense peaks. Data were analyzed using Xcalibur (Thermo Fisher Scientific; version 2.2 SP1.48) and Free Style TM 1.8 SP2 QF1 (Thermo Scientific).

Absolute quantification of the compounds was performed by HPLC on a Thermo Ultimate 3000 (Thermo Fisher Scientific) using an identical chromatographic procedure as described above. Compounds were detected by a DAD detector (260 nm; Thermo Fisher Scientific). Isorhamnetin-3-O-glucoside, kaempferol-3-O-glucoside, isoquercitrin and rutin (all Extrasynthese), narcissin (Targetmol Chemicals Inc., Boston, MA, USA), kaempferol-3-O-β rutinosid, and caffeic acid (both Sigma Aldrich), quercetin-3-O-(6″-O-malonyl)-β-D-glucosid (THP Medical Products, Vienna, Austria) were used as standards for calibration.

### 2.7 Statistical analysis

Statistical analysis and data visualization were conducted using GraphPad Prism (Version 11.0.0; GraphPad Software Inc., CA, USA). For the biofilm and swarming motility data, the assumptions for one-way ANOVA were assessed by testing normality using the Shapiro-Wilk test and homogeneity of variances using the Brown-Forsythe test. The Brown-Forsythe test indicated heterogeneity of variances in some datasets, and the residuals deviated from normality in all datasets. Therefore, a nonparametric Kruskal-Wallis test followed by Dunn’s multiple comparison test was performed to compare treatment groups with the untreated control. Data are presented as mean ± SD, unless otherwise stated in the figure legends. A *p-*value ≤ 0.05 was considered statistically significant and indicated as *p ≤ 0.05, **p ≤ 0.01, ***p ≤ 0.001 or ****p ≤ 0.0001.

The graphical abstract was created in BioRender (Science Suite Inc., Toronto, ON, Canada). Photos and other images were processed in CorelDRAW^®^ Graphics Suite (Version 21.0.0.593; Corel Corporation, Ottawa, ON, Canada).

## 3 Results

### 3.1 Antimicrobial activity of extracts

200 aqueous plant extracts prepared at 2 different extraction temperatures, termed “cold/hot extracts,” were screened for their antimicrobial activity against three Gram-negative pathogens (*E. coli*, *S.* Typhimurium and *P. aeruginosa*), three Gram-positive pathogens (*S. aureus*, *S. mutans* and *E. faecalis*) and three non-pathogenic lactobacilli (*L. paracasei*, *L. plantarum* and *L. rhamnosus*). The pH of the extracts was not adjusted prior to antimicrobial testing, as the objective was to evaluate the extracts in their native form. All screened extracts, their used plant part, the site and date of plant material collection as well as the pH, dry matter content, TPC and antioxidant activity determined by FRAP assay, are listed in the Supplementary Table 1.

For the initial screening, antimicrobial activity against at least one tested pathogen was detected for 21 extracts, including *Allium sativum*, *Allium ursinum*, *Apium graveolens*, *Castanea sativa*, *Eruca vesicaria* subsp. *sativa*, *Fragaria × ananassa*, *Mentha spicata*, *Origanum vulgare*, *Ribes × nidigrolaria*, *Rubus fruticosus* and *Rubus laciniatus*. All plant extracts showing antimicrobial activity in the disk diffusion assay, along with their common names, their used plant parts and characteristics, are listed in Table 1. In 9 plant species, both hot and cold extracts showed antimicrobial activity in the disk diffusion assay. Only 3 plant species, *Eruca vesicaria* subsp. *sativa*, *Mentha spicata* and *Rubus fruticosus*, were active against at least one pathogenic strain exclusively in hot extracts. In contrast to pathogens, none of the tested lactobacilli was susceptible to any of these 21 plant extracts in the disk diffusion assay.

**TABLE 1:** Plant extracts showing antimicrobial activity in the disk diffusion assay, including species, common name, plant part, pH of the extract, TPC [mg GAE per g dry weight of the extract] and dry extract concentration [mg/mL].

Genus/species	Common name	Plant part	pH	TPC [mg GAE/g dry weight]	Dry extract concentration [mg/mL]
*Allium sativum* (1)	Garlic	Tuber	6.381 ± 0.004	2.29 ± 0.13	62.67 ± 10.40
*Allium sativum* (2)	Garlic	Tuber	6.236 ± 0.006	1.91 ± 0.10	64.00 ± 7.48
*Allium ursinum* (1)	Wild garlic	Leaves	5.993 ± 0.002	12.84 ± 0.17	28.33 ± 0.70
*Allium ursinum* (2)	Wild garlic	Leaves	5.770 ± 0.002	9.94 ± 0.20	24.40 ± 3.28
*Apium graveolens* (1)	Celeriac	Tuber	5.610 ± 0.002	2.43 ± 0.21	31.00 ± 0.87
*Apium graveolens* (2)	Celeriac	Tuber	5.436 ± 0.003	1.81 ± 0.04	25.60 ± 1.11
*Castanea sativa* (1)	Sweet chestnut	Leaves	4.904 ± 0.002	15.82 ± 0.09	7.73 ± 0.31
*Castanea sativa* (2)	Sweet chestnut	Leaves	4.840 ± 0.002	23.08 ± 0.27	9.67 ± 0.50
*Eruca vesicaria* subsp. *sativa* (2)	Rocket / arugula	Leaves	5.340 ± 0.001	5.65 ± 0.09	21.87 ± 1.22
*Fragaria* × *ananassa* (1)	Strawberry	Fruit	3.362 ± 0.002	17.92 ± 0.08	49.73 ± 2.90
*Fragaria* × *ananassa* (2)	Strawberry	Fruit	3.367 ± 0.001	19.16 ± 0.73	47.73 ± 1.33
*Mentha spicata* (2)	Spearmint	Leaves	6.283 ± 0.003	51.37 ± 2.83	22.27 ± 0.80
*Origanum vulgare* (1)	Pink-flowering oregano	Blossom	5.517 ± 0.002	85.02 ± 1.81	17.33 ± 1.81
*Origanum vulgare* (2)	Pink-flowering oregano	Blossom	5.521 ± 0.002	88.29 ± 2.13	15.67 ± 1.29
*Origanum vulgare* (1)	White-flowering oregano	Blossom	5.328 ± 0.004	124.20 ± 3.79	19.00 ± 0.35
*Origanum vulgare* (2)	White-flowering oregano	Blossom	5.332 ± 0.001	95.06 ± 2.54	18.40 ± 0.60
*Ribes* × *nidigrolaria* (1)	Jostaberry	Leaves	5.319 ± 0.003	41.00 ± 2.27	16.80 ± 0.35
*Ribes* × *nidigrolaria* (2)	Jostaberry	Leaves	5.501 ± 0.002	45.40 ± 0.83	15.33 ± 0.42
*Rubus fruticosus* (2)	Blackberry	Fruit	3.226 ± 0.003	20.86 ± 0.78	41.67 ± 2.05
*Rubus laciniatus* (1)	Cutleaf evergreen blackberry	Leaves and stem	5.139 ± 0.003	21.24 ± 0.19	27.13 ± 0.23
*Rubus laciniatus* (2)	Cutleaf evergreen blackberry	Leaves and stem	5.161 ± 0.002	28.78 ± 0.15	11.87 ± 0.50

Data is shown as mean ± SD of three measurements. Numbers in parentheses following each extract name denote the extraction type: (1) cold extract (2) hot extract.

**FIGURE 1:**
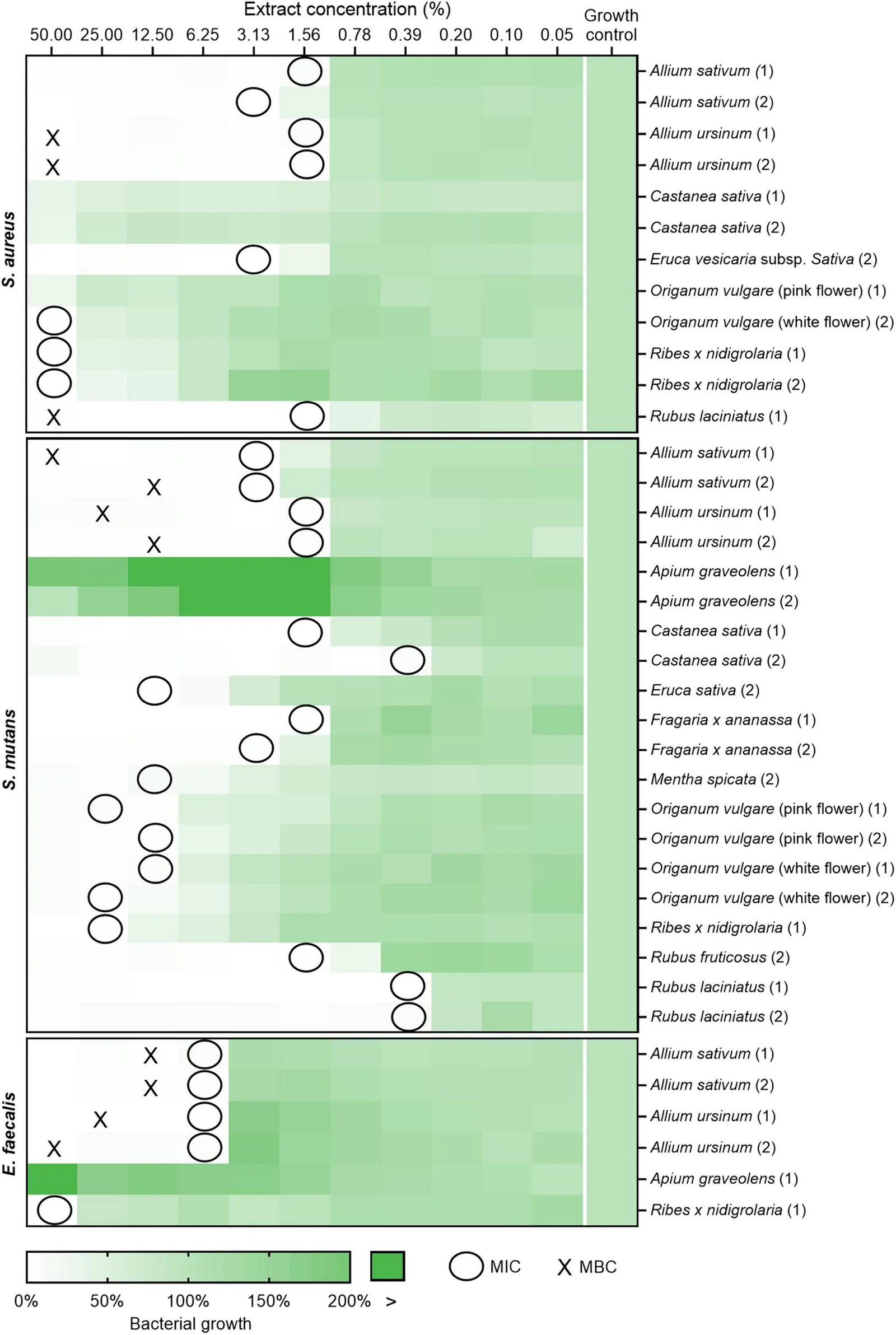
Minimum inhibitory concentrations (MIC) and minimum bactericidal concentrations (MBC) of plant extracts against the following Gram-positive bacteria: *S. aureus*, *S. mutans* and *E. faecalis*. Heatmaps illustrate bacterial growth across decreasing extract concentrations (50%–0.05%). The MIC is marked by a circle and the MBC by an X at the corresponding concentration. For each bacterial strain, the untreated culture served as the growth control. Numbers in parentheses following each extract name denote the extraction type: (1) cold extract (2) hot extract.

**FIGURE 2:**
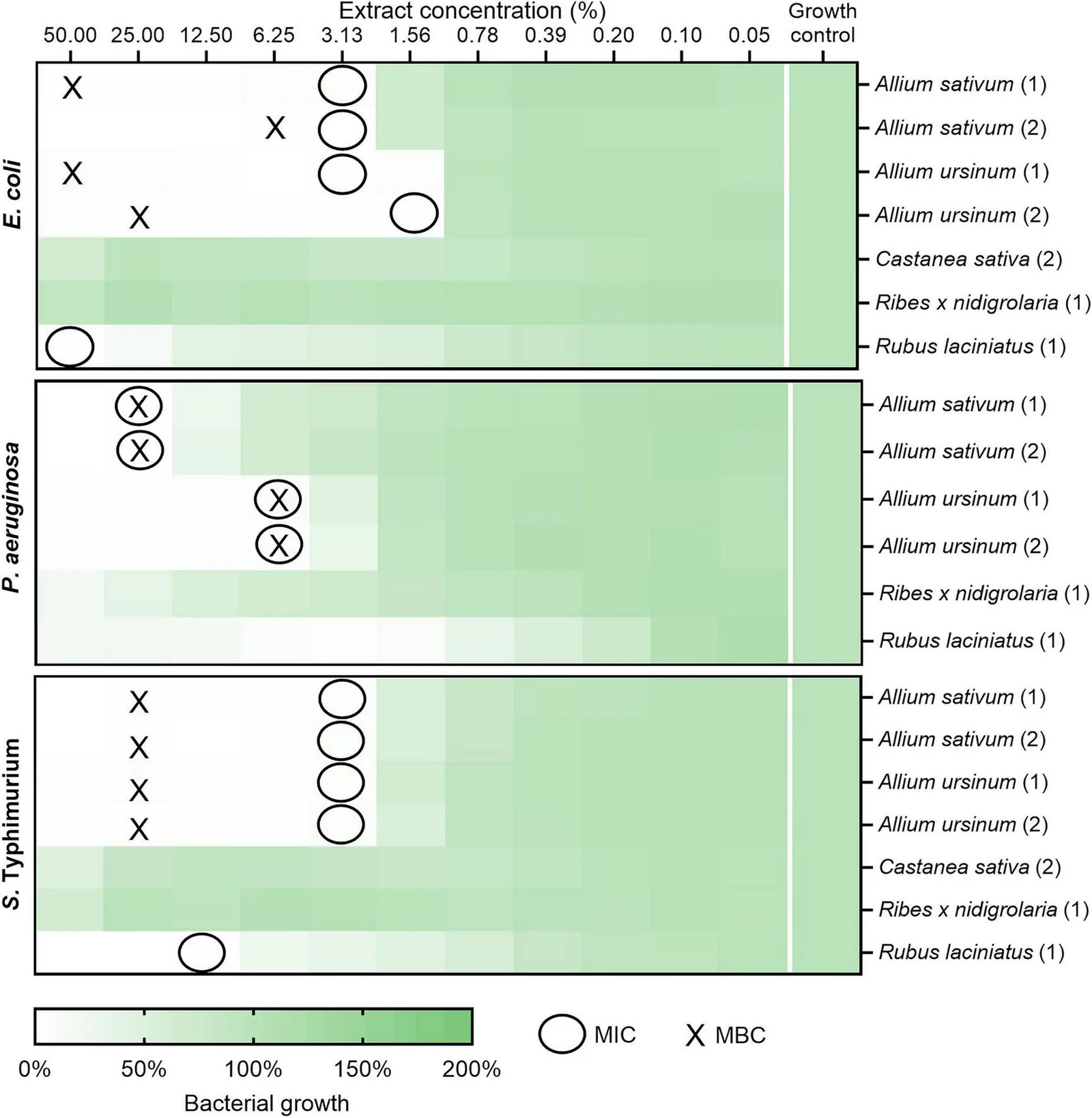
Minimum inhibitory concentrations (MIC) and minimum bactericidal concentrations (MBC) of plant extracts against the following Gram-negative bacteria: *E. coli*, *S.* Typhimurium and *P. aeruginosa*. Heatmaps illustrate bacterial growth across decreasing extract concentrations (50%–0.05%). The MIC is marked by a circle and the MBC by an X at the corresponding concentration. For each bacterial strain, the untreated culture served as the growth control. Numbers in parentheses following each extract name denote the extraction type: (1) cold extract (2) hot extract.

Next, the MIC and MBC values of the 21 positive hits were determined against the sensitive pathogens by the broth microdilution method. Although inhibition was not observed for all pathogens in the disk diffusion assay, the MIC and MBC values of *Allium sativum* and *Allium ursinum* extracts were tested against all pathogens because of their well-known antibacterial activity (Gull et al., 2012; Stupar et al., 2022). All MIC and MBC values are listed in Supplementary Table 2 (cold extracts) and Supplementary Table 3 (hot extracts) as % (v/v) and as mg dry extract per mL, along with the pH values of the respective extracts diluted in MH2 medium. The pH values of extracts diluted in MH2 medium at the highest tested concentration exceeded 6.0, with the exception of the berry extracts, which showed pH values of approximately 4.2. In summary, 19 extracts showed defined MIC values (concentrations between 0.39 and 50%), while only 5 extracts exhibited bactericidal activity (concentrations between 6.25 and 50%). Although extracts from *Apium graveolens* produced clear inhibition zones against Gram-positive bacteria in the disk diffusion assay, no antibacterial activity was detected in the microdilution assay. On the contrary, the *Apium graveolens* extracts appeared to promote bacterial growth under the microdilution conditions. Importantly, the growth-promoting effect was not an artifact caused by absorbance of the extract itself because an extract-specific blank correction was performed for all measurements. The discrepancy between the disk diffusion and microdilution assay may be explained by the different principles of the methods. While the disk diffusion assay depends on compound diffusion in agar, the microdilution assay evaluates bacterial growth in a homogenous liquid environment. Since the microdilution assay was limited to a maximum test concentration of 50%, plant extracts, such as *Apium graveolens*, that showed activity in the disk diffusion assay, where they were applied undiluted, but not in the microdilution assay, likely require higher concentrations to exert inhibitory and bactericidal activities. The results of the microdilution assay for each pathogen are summarized in form of heatmaps in Figure 1 (Gram-positive strains) and Figure 2 (Gram-negative strains).

Our findings demonstrate that Gram-positive bacteria, in particular *S. mutans* and *S. aureus*, were more susceptible to plant extracts than Gram-negative bacteria. *S. mutans* proved to be the most susceptible strain among the bacteria tested. Extracts derived from *Rubus fruticosus*, *Fragaria × ananassa*, *Mentha spicata* and *Castanea sativa* were active on *S. mutans*, whereas the other pathogens did not show comparable susceptibility to these plant extracts. Although growth of *S. mutans* was inhibited, a bactericidal effect was rarely observed, with MBCs detected only for *Allium sativum* and *Allium ursinum* extracts. In contrast to the other pathogens, the Gram-negative species *P. aeruginosa* was inhibited exclusively by extracts derived from *Allium sativum* and *Allium ursinum*. For *P. aeruginosa*, the MIC values were identical to the MBC values, indicating that growth inhibition occurred at bactericidal concentrations. Beyond *Allium sativum* and *Allium ursinum*, cold leaf extracts from *Ribes × nidigrolaria* and *Rubus laciniatus* also exhibited notable inhibitory effects against several pathogens. *Ribes × nidigrolaria* extracts inhibited all Gram-positive strains, whereas *Rubus laciniatus* extracts were active against all strains except *P. aeruginosa* and *E. faecalis*.

### 3.2 Anti-biofilm activity of extracts

Biofilm formation assays were performed with all pathogenic strains except *P. aeruginosa* and *E. faecalis*. For these two species, biofilm quantification did not provide sufficiently robust and reproducible results despite extensive optimization of assay conditions and validation using literature-reported biofilm inhibitors. Therefore, these data were excluded from the final analysis, although the corresponding raw data are available in the Supplementary Table 4 (*S.* Typhimurium) and Supplementary Table 5 (*P. aeruginosa*). Reproducible measurements were obtained for the remaining pathogens. Based on the criteria proposed by Stepanović et al. (2007), the bacterial strains used in this study exhibited moderate (*S. mutans* and *E. coli*) to strong (*S. aureus* and *S.* Typhimurium) biofilm-forming capacities. To allow the assessment of biofilm-specific effects rather than growth inhibition, plant extract concentrations used in the biofilm assays were selected to maintain ≥80% growth relative to the untreated control in preliminary cytotoxicity assays performed in the respective biofilm-promoting medium. The observation that some concentrations required in the biofilm assay exceeded the previously determined MIC values may be attributed to differences in media compositions between the assays as well as the protective nature of biofilms against harsh environmental conditions.

**FIGURE 3:**
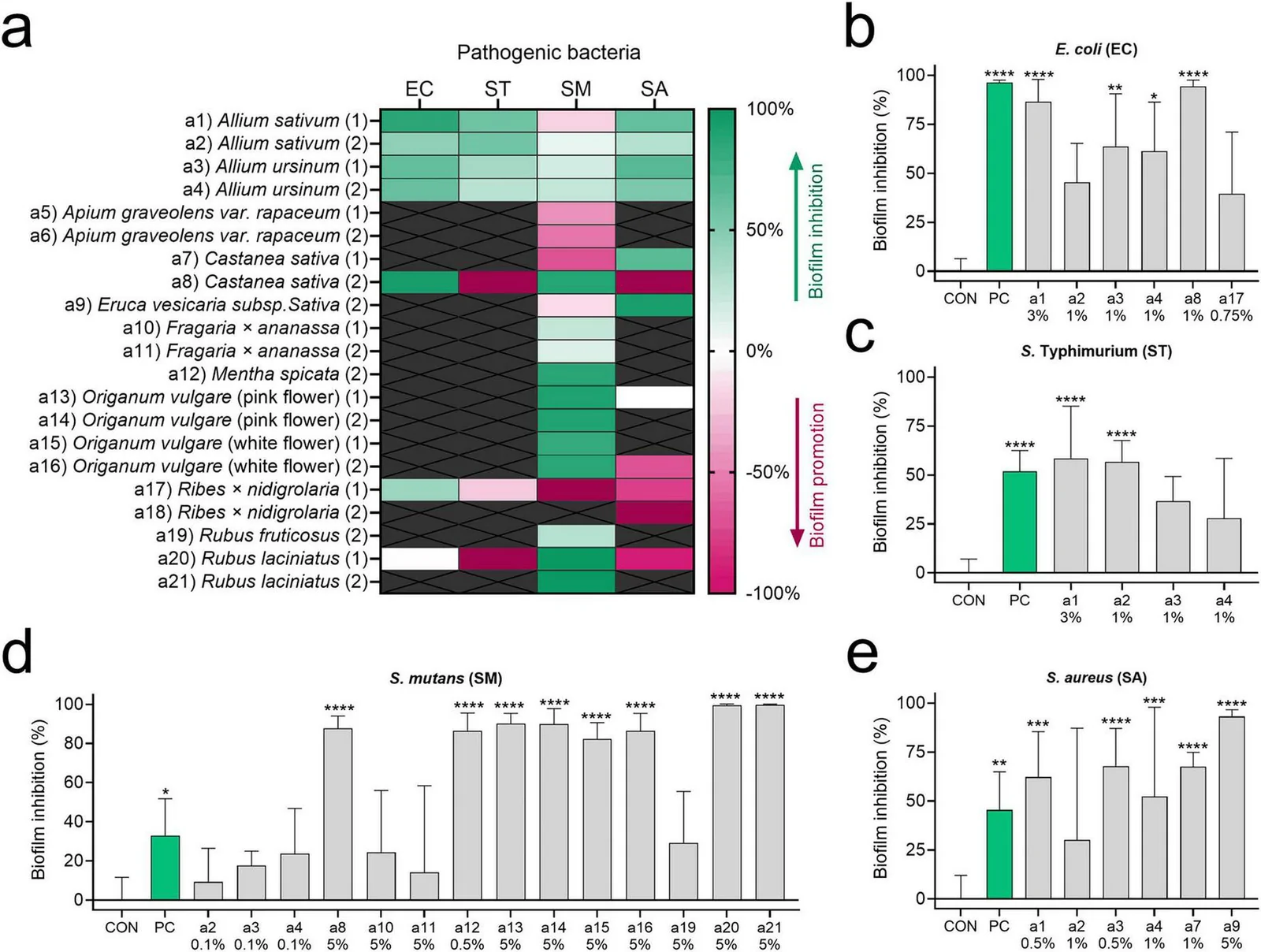
Effect of plant extracts on biofilm formation of pathogens. **(a)** Heatmaps showing the relative reduction of biofilm biomass, expressed as percentage inhibition. Positive values indicate biofilm inhibition (green), whereas negative values indicate biofilm promotion (pink). Numbers in parentheses following each extract name denote the extraction type: (1) cold extract (2) hot extract. Extracts exhibiting anti-biofilm activity are further presented in bar charts as mean ± SD of three independent experiments: **(b)** Anti-biofilm effects against *E. coli*; quercetin (20 μg/mL) served as positive control. **(c)** Anti-biofilm effects against *S.* Typhimurium; phloretin (100 μg/mL) served as positive control. **(d)** Anti-biofilm effects against *S. mutans;* xylitol (2.5%) served as positive control. **(e)** Anti-biofilm effects against *S. aureus*; phloretin (10 μg/mL) served as positive control. Statistical analysis was performed for each strain using nonparametric Kruskal-Wallis test with Dunn’s *post hoc* multiple comparisons versus untreated control. CON, untreated growth control; PC, positive control. Significance: *p ≤ 0.05, **p ≤ 0.01, ***p ≤ 0.001 or ****p ≤ 0.0001.

The effects of the extracts on biofilm formation in the tested pathogens are summarized in the heatmap shown in Figure 3a. Overall, the extracts displayed species-specific and, in some cases, opposing activities. Several plant extracts reduced biofilm formation in certain bacteria (Figures 3b–e) while promoting it in others. For instance, the hot *Castanea sativa* extract clearly decreased biofilm formation in *S. mutans* by 88% and *E. coli* by 94%, whereas an increase was observed in *S.* Typhimurium and *S. aureus*. A similar pattern was detected for the cold *Rubus laciniatus* extract, which fully suppressed biofilm formation in *S. mutans* but stimulated it in *S.* Typhimurium and *S. aureus*. Cold *Ribes × nidigrolaria* extract enhanced biofilm production in Gram-positive species but showed anti-biofilm activity in *E. coli* (reduction by 40%). Nevertheless, extracts from *Allium sativum* and *Allium ursinum* showed consistent anti-biofilm activity across all tested strains, except for the cold *Allium sativum* extract, which exhibited no detectable effect on Gram-positive bacteria. Hot *Mentha spicata* extract as well as all *Origanum vulgare* extracts inhibited biofilm formation in *S. mutans* by 82%–90%. In addition, hot white-flower *Origanum vulgare* extract also reduced *S. aureus* biofilm formation by 47%. Berry extracts were active only against *S. mutans* biofilms with a biofilm reduction of 24% for cold *Fragaria × ananassa* extract, 14% for hot *Fragaria × ananassa* extract and 29% for hot *Rubus fruticosus* extract.

### 3.3 Inhibition of swarming motility

Swarming motility assays were performed with *P. aeruginosa* (Yang et al., 2017) and *S.* Typhimurium (Partridge and Harshey, 2023), as these species exhibit well-characterized swarming motility on semi-solid agar plates. In contrast, the other pathogenic strains used in this study are non-motile or do not exhibit reproducible swarming behavior under standard laboratory conditions.

**FIGURE 4:**
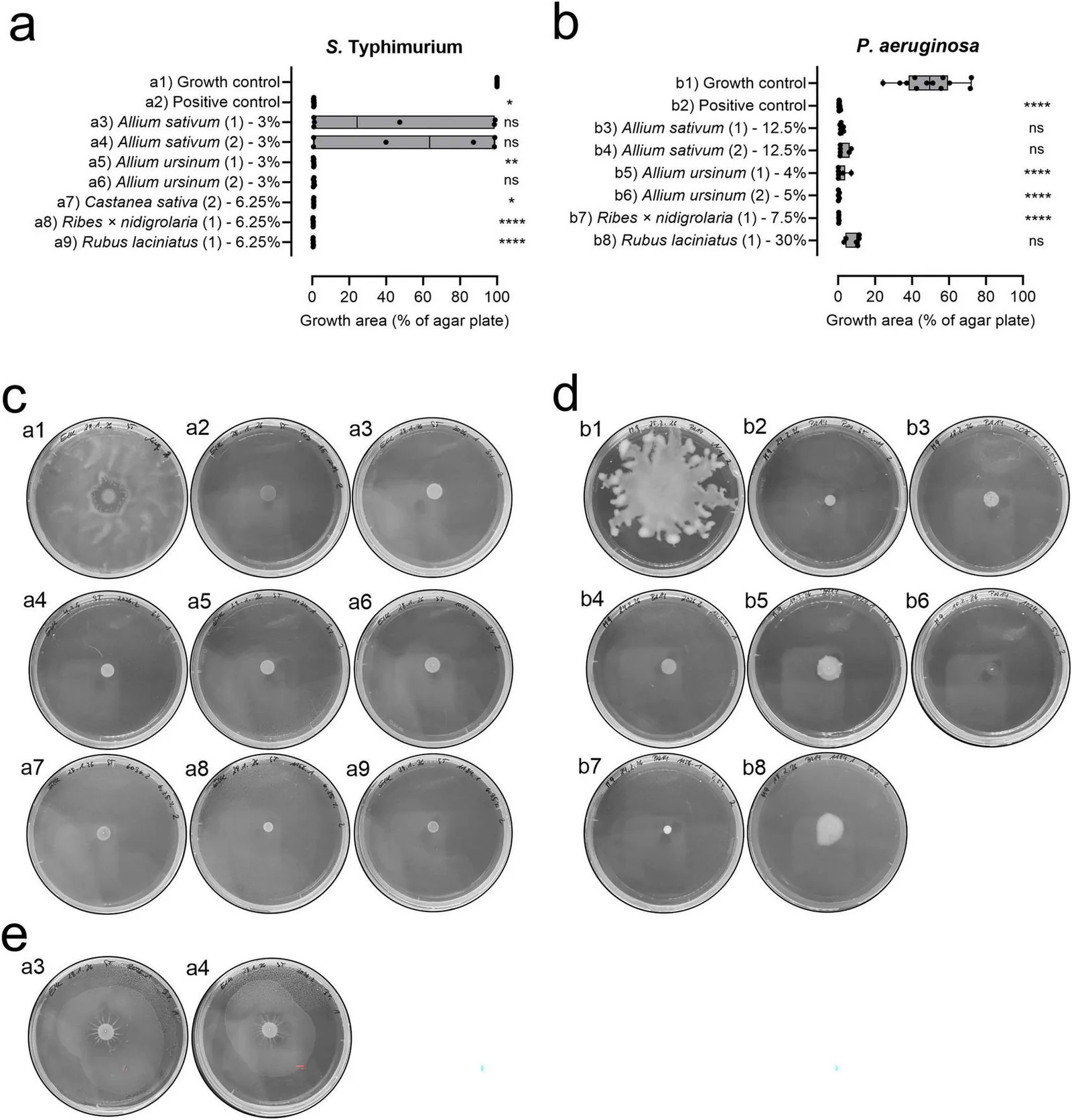
Swarming motility of panel **(a)** *S*. Typhimurium (ST) and **(b)** *P. aeruginosa* (PA) in the presence of plant extracts. Swarming activity is expressed as the percentage of the agar plate covered by bacterial growth. The growth area was determined by image analysis and calculated as the area occupied by bacterial spreading relative to the total area of the agar plate. Data are shown as boxplots with each data point corresponding to one agar plate (three independent experiments, each performed in duplicate). The concentrations tested were selected from a range of extract concentrations and represent those that did not inhibit bacterial growth while effectively reducing biofilm formation. Untreated cultures served as growth controls, and sodium salicylate (ST: 15 mM; PA: 35 mM) served as positive control. Numbers in parentheses following each extract name denote the extraction type: (1) cold extract (2) hot extract. Representative images of swarming behavior are shown in panel **(c)** for *S.* Typhimurium on Eiken agar plates and **(d)** for *P. aeruginosa* on M9 agar plates. **(e)** Representative images illustrating that *Allium sativum* extracts did not consistently suppress swarming motility of *S.* Typhimurium at a concentration of 3%. Statistical analysis was performed for each strain using nonparametric Kruskal-Wallis test with Dunn’s *post hoc* multiple comparisons versus growth control. ns, not significant. Significance: *p ≤ 0.05, **p ≤ 0.01, or ****p ≤ 0.0001.

The concentrations of extracts derived from *Allium sativum*, *Allium ursinum*, *Castanea sativa*, *Ribes × nidigrolaria* and *Rubus laciniatus* that inhibited swarming without impairing bacterial growth are shown in Figure 4. The lowest effective concentrations were observed for *Allium sativum* and *Allium ursinum* in *S.* Typhimurium. Notably, 3% of *Allium sativum* did not lead to consistent results, completely blocking swarming in some experiments (Figure 4c) while only slightly reducing it in others (Figure 4e). For *P. aeruginosa*, higher concentrations of *Allium ursinum* (4%–5%) were required to inhibit swarming. *Castanea sativa* inhibited swarming of *S.* Typhimurium at 6.25%, whereas extracts from *Ribes × nidigrolaria* and *Rubus laciniatus* displayed anti-swarming activity in both species, but at different concentrations. In general, *P. aeruginosa* required higher extract concentrations to achieve complete swarming inhibition, which was also reflected in the positive control. All extract concentrations that were tested in the swarming motility assay are summarized in Supplementary Tables 6 (*S.* Typhimurium) and Supplementary Table 7 (*P. aeruginosa*).

### 3.4 Cytotoxicity assessment of selected plant extracts in Caco-2 cells

To obtain a first indication of the safety profile of the positive hits, cell viability was evaluated in human Caco-2 cells. As shown in Supplementary Table 8, all tested extracts (1%) maintained cell viability above 90%, indicating no cytotoxicity under the applied conditions.

### 3.5 Phytochemical composition of *Ribes × nidigrolaria* leaf extract

**FIGURE 5:**
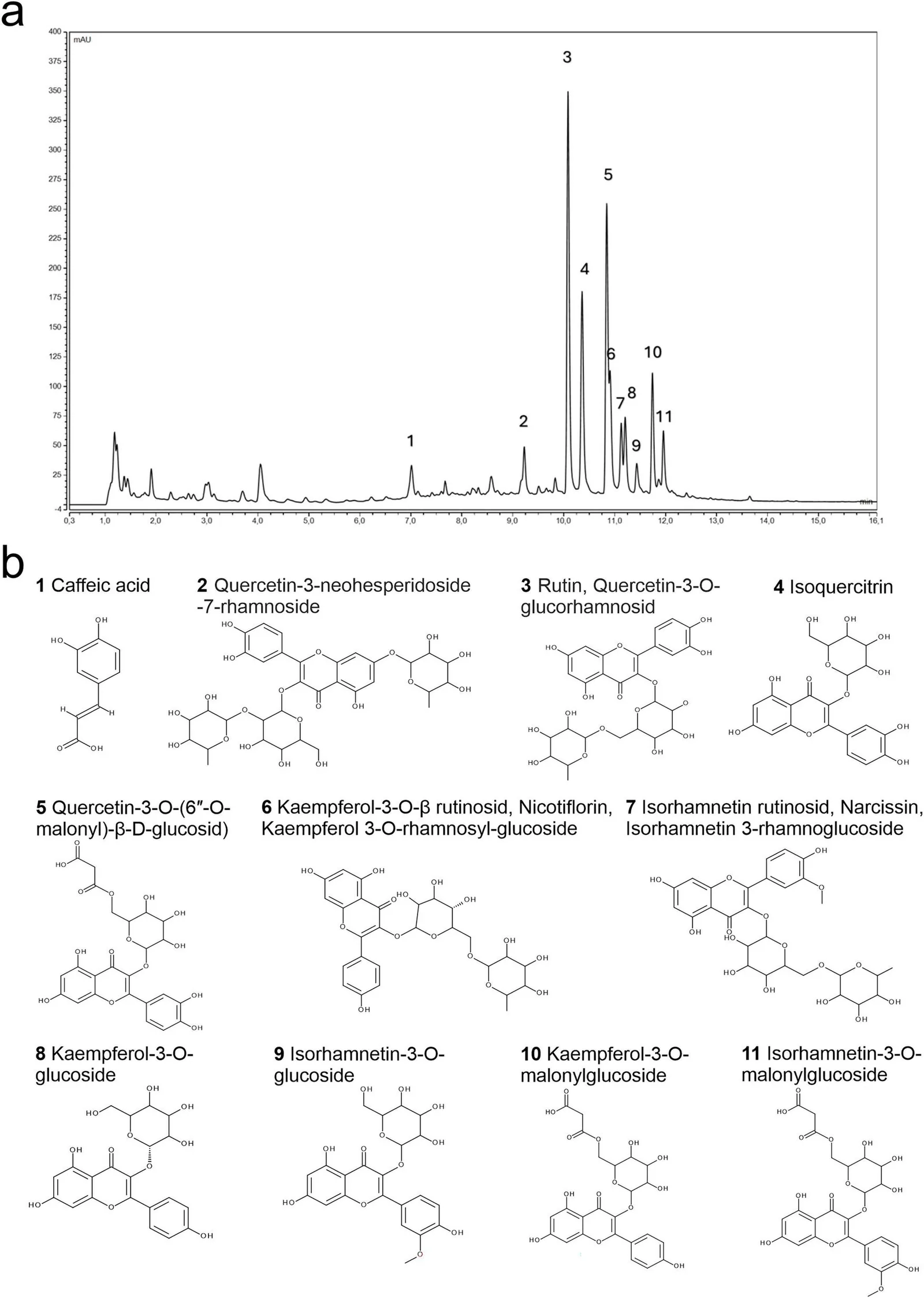
Representative HPLC analyses of *Ribes × nidigrolaria* leaf extract. **(a)** HPLC chromatogram: 1 caffeic acid; 2 quercetin-3-neohesperidoside-7-rhamnoside; 3 rutin; 4 isoquercitrin; 5 quercetin-3-*O*-(6″-O-malonyl)-β-D-glucosid; 6 nicotiflorin; 7 narcissin; 8 kaempferol-3-*O*-glucoside; 9 isorhamnetin-3-*O*-glusoside; 10 kaempferol-3-*O*-malonylglucoside; 11 isorhamnetin-3-*O*-malonylglucoside. (b) Structural formulas created using Draw© software from Dassault Systčmes BIOVIA.

Potential active compounds present in *Ribes × nidigrolaria* leaf extract, which showed previously unreported extract-level activities, were further investigated using LC-MS/MS. This extract was selected for detailed analysis because the phytochemical composition of *Ribes × nidigrolaria* leaves has not yet been characterized, whereas the constituents of other antibacterial plant extracts, such as *Allium sativum* (Bar et al., 2022), *Allium ursinum* (Hangweirer et al., 2025), *Castanea sativa* (Formato et al., 2022; Silva et al., 2020) and *Rubus laciniatus* (Memete et al., 2023; Stintzing et al., 2002), are already described in literature. Accordingly, the objective was not to conduct an untargeted metabolomic profiling but to identify characteristic constituents of the promising *Ribes × nidigrolaria* leaf extract. As shown in the HPLC chromatogram (Figure 5), the main peaks were tentatively annotated and most of them were validated using standards. The majority of identified compounds were glycoside derivates of quercetin (peaks 2, 3, 4, 5), kaempferol (peaks 6, 8, 10) and isorhamnetin (peaks 7, 9, 11). In addition, caffeic acid was identified (peak 1). Quantification revealed the highest concentrations for rutin (284 μg/mL), followed by quercetin-3-O-(6″-O-malonyl)-β-D-glucosid (214.5 μg/mL), isoquercitrin (162.5 μg/mL), nicotiflorin (91.5 μg/mL), kaempferol-3-O-glucoside (56.7 μg/mL), narcissin (48.8 μg/mL), caffeic acid (38.5 μg/mL), and isorhamnetin-3-O-glucoside (17.9 μg/mL). A detailed overview of all tentatively identified compounds is provided in Supplementary Table 9. To facilitate comparison of the phytochemical composition of the most active extracts, representative HPLC-DAD fingerprints and m/z signals are provided in the Supplementary Information (Supplementary Figures 1–5 and Supplementary Table 10).

## 4 Discussion

As antibiotic resistance continues to pose a major global challenge, the search for effective alternatives to conventional antibiotics is of utmost importance. Plant extracts with antimicrobial constituents might represent promising alternatives for the control of pathogens associated with foodborne infections and could also be incorporated into food or feed to inhibit or delay the growth of harmful bacteria. The multitude of bioactive substances, such as polyphenols (Lobiuc et al., 2023), present in these extracts enables a broad spectrum of antimicrobial mechanisms that cannot be replicated by a single synthetic compound (Oulahal and Degraeve, 2021). Moreover, it has been reported that many plant extracts and plant secondary metabolites can exhibit anti-virulence activity, such as swarming or biofilm formation inhibition, without affecting bacterial viability. Even if such plant extracts do not directly kill pathogenic bacteria, they may still contribute to the control of bacterial infections by targeting virulence traits and potentially reducing the development of resistance (Díaz-Nuñez et al., 2021). In this study, we tested 200 aqueous plant extracts for their antibacterial, anti-biofilm and anti-swarming activities against clinically relevant pathogens, including Gram-negative and Gram-positive bacteria, which differ in their pathogenicity and clinical significance to capture a broad spectrum of activity. The extracts were obtained from well-known plant species native to, or commonly occurring in, Upper Austria and bordering regions. In addition to their regional availability, plant species were selected based on their accessibility in compliance with the Nagoya Protocol and their suitability for future applications as they are generally considered edible and non-toxic.

Notably, the aqueous extracts of *Allium sativum* and *Allium ursinum* exhibited strong antibacterial activity against all tested pathogens, which is consistent with previous studies (Gull et al., 2012; Stupar et al., 2022). This may be explained by the high water solubility of many bioactive sulfur-containing compounds present in *Allium sativum* (Ruddock et al., 2005), which facilitates their extraction into aqueous solutions. Although allicin is the best-known antimicrobial substance in *Allium sativum*, it is unlikely to be found in high quantities in the aqueous extracts, as the molecule is very unstable and degrades into products such as diallyl-sulfide, diallyl-disulfide, diallyl-trisulfide, allyl methyl sulfide and ajoene (Bhatwalkar et al., 2021). Recently, our research group also reported on the beneficial effects of an *Allium ursinum* extract on *Caenorhabditis elegans* infected with *P. aeruginosa* and identified kaempferol 3-*O*-neohesperidoside, allyl methyl disulfide, diallyl-sulfide and diallyl-disulfide as the main active compounds (Hangweirer et al., 2025).

The antimicrobial activity of *Mentha spicata* and *Origanum vulgare* essential oils against a variety of microorganisms has been widely described (Gavaric et al., 2015; Saba et al., 2024). Since essential oils are known to have limited water solubility and are therefore present at lower concentrations in aqueous extracts, previous studies have reported enhanced antimicrobial activity for ethanolic extracts of *Origanum vulgare* compared to aqueous extracts (Idir et al., 2022). In the present study, however, aqueous extracts of *Mentha spicata* and *Origanum vulgare* still demonstrated inhibitory effects against *S. aureus* and *S. mutans*, highlighting their antimicrobial potential even in water-based systems. Despite solubility-related limitations, the use of water as an extraction solvent offers several important advantages. Its non-toxic nature, safety and ease of handling make it particularly suitable for use in food-related applications (Plaskova and Mlcek, 2023). Furthermore, water as solvent offers excellent compatibility with a wide range of biological assays as it does not affect bacterial growth. Consequently, this allows observed antimicrobial or anti-virulence effects to be directly attributed to the bioactive constituents of the extracts, thereby strengthening the reliability and applicability of the findings. In line with this, our results suggest that the extraction temperature appears to have a smaller impact on antimicrobial activity than the plant species, as aqueous extracts from the same species were active in both hot and cold extracts.

Our observation that Gram-positive pathogens, except for *E. faecalis*, react more sensitively to the tested plant extracts than Gram-negative bacteria can be partly explained by structural differences in the cell wall of the bacteria. The outer membrane of Gram-negative bacteria limits the penetration of numerous antimicrobial agents, especially of large, charged molecules (Galdiero et al., 2012). The comparatively low susceptibility of the Gram-positive bacterium *E. faecalis* observed in this study is supported by previous reports (Macambira et al., 2024). This phenomenon might be attributed to the remarkable adaptability of *E. faecalis* and its resistance to a broad range of antimicrobial agents (Ali et al., 2017). For instance, *E. faecalis* possesses ATP-binding cassette (ABC) multidrug efflux pumps that actively remove antimicrobial compounds, thereby limiting their intracellular accumulation (Lee et al., 2003). Furthermore, we found none of the tested *Lactobacillus* strains to be sensitive against plant extracts in the disk diffusion assay, which is in agreement with previous studies on the tolerance of lactic acid bacteria to plant-derived antimicrobials (Nakayama et al., 2015). This selective resistance is of practical relevance as it indicates that certain plant extracts could be employed to specifically suppress pathogenic microorganisms while preserving probiotic lactic acid bacteria (Oulahal and Degraeve, 2021). Furthermore, the absence of relevant cytotoxic effects in Caco-2 cells under the tested conditions supports their potential suitability for food and feed applications.

In addition to assessing the antibacterial activity of plant extracts, we also examined their influence on biofilm formation. Interestingly, reduction in biofilm formation of *S. mutans*, a key cariogenic pathogen (Lemos et al., 2019; Lin et al., 2021) was detected for several plant extracts of *Castanea sativa, Mentha spicata*, blossom extracts of *Origanum vulgare* as well as extracts of *Rubus laciniatus* exhibiting the strongest biofilm reducing effects. To our knowledge, this is the first study to report antibacterial and anti-biofilm activity of *Rubus laciniatus* and *Castanea sativa* leaf extracts against *S. mutans*. Previous studies only demonstrated antimicrobial effects of *Castanea sativa* leaf extracts against other Gram-positive bacteria such as *S. aureus* (Quave et al., 2015; Silva et al., 2020). The observed effects are likely attributable to the plant-derived compounds present in the extracts which may interfere with biofilm production. In this context, Silva et al. (2020) characterized ethanolic extracts from different parts of *Castanea sativa*, including shell, inner shell, bur and leaves, and reported the highest phenolic content in leaf extracts with Trigalloyl-HHDP-glucose identified as the main compound. In addition, *Castanea sativa* leaves are known to contain various flavonoid derivates of quercetin, kaempferol and isorhamnetin, as well as gallo- and ellagitannins (Formato et al., 2022; Silva et al., 2020). Our observation that fruit extracts of *Fragaria × ananassa* and *Rubus fruticosus* also inhibit biofilm formation in *S. mutans* is consistent with previous reports indicating that berry extracts rich in tannins and flavonoids, particularly proanthocyanidins, can interfere with biofilm formation (Philip et al., 2019). One proposed mechanism underlying the anti-biofilm effect in *S. mutans* involves the inhibition of glycosyltransferases, key enzymes in the synthesis of extracellular polysaccharides such as glucans, which are essential for *S. mutans* biofilm development (Ren et al., 2016). However, the underlying mechanisms were not investigated in the present study.

The pathogenic strains *S.* Typhimurium (Partridge and Harshey, 2023) and *P. aeruginosa* (Yang et al., 2017) are well-known for their ability to swarm on semi-solid agar plates. There is evidence that different plant extracts and plant-derived compounds, such as citrus extracts and flavanones (Barbosa et al., 2021), cranberry proanthocyanidins (O’May and Tufenkji, 2011) as well as phenolic acids (Ugurlu et al., 2016) can inhibit swarming motility of *S.* Typhimurium and *P. aeruginosa*. In the present study, we demonstrated that extracts derived from *Allium sativum*, *Allium ursinum*, *Castanea sativa*, *Ribes × nidigrolaria* and *Rubus laciniatus* suppressed swarming motility without completely inhibiting bacterial growth. To the best of our knowledge, no study has previously reported on the ability of leaf extracts derived from *Castanea sativa*, *Ribes × nidigrolaria* and *Rubus laciniatus* to inhibit the swarming of these two pathogen species.

In addition to well-established antimicrobial extracts such as those of *Allium sativum* and *Allium ursinum*, we also identified several previously unreported plant extracts with notable effects, particularly leaf extracts of *Rubus laciniatus* and *Ribes × nidigrolaria*. The former, *Rubus laciniatus* leaf extract, was active against Gram-positive and Gram-negative bacteria, suppressed *S. mutans* biofilms and reduced the swarming motility of *P. aeruginosa* and *S.* Typhimurium. To date, no other studies have reported antimicrobial properties for *Rubus laciniatus*. However, closely related species, including *Rubus idaeus* (raspberry) and *Rubus fruticosus* (blackberry), have been shown to possess antibacterial activities in their leaf extracts (Kucharski et al., 2022). Phytochemical analyses of various blackberry cultivars, including *Rubus laciniatus*, indicate that blackberries are generally rich in anthocyanins, particularly cyanidin derivates, as well as flavonols, epicatechin and ellagic acid derivates (Memete et al., 2023; Stintzing et al., 2002). Consistently, blackberry leaf extracts have also been described to contain flavonols, epicatechin and ellagic acid (Paczkowska-Walendowska et al., 2021).

The antibacterial activity of *Ribes × nidigrolaria*, which is a hybrid of black currant (*Ribes nigrum*) and gooseberry (*Ribes uva-crispa*) (Hempfling et al., 2013), has been described in a single study, focusing on fruit extracts against different bacterial species (Bulgaru et al., 2024), whereas no studies on the leaves have been reported to date. Here, we could show that *Ribes × nidigrolaria* leaf extracts inhibited growth of Gram-positive bacteria but also significantly reduced biofilm formation in Gram-negative *E. coli.* In addition, swarming of *P. aeruginosa* and *S.* Typhimurium was significantly suppressed by cold *Ribes × nidigrolaria* leaf extracts at 7.5% and 6.25%, respectively, suggesting that they may also affect Gram-negative bacteria by targeting virulence-associated traits rather than directly inhibiting bacterial growth. Importantly, this study investigated the phytochemical composition of *Ribes × nidigrolaria* leaves for the first time. Caffeic acid as well as derivates of quercetin, kaempferol and isorhamnetin were identified as the main components in aqueous *Ribes × nidigrolaria* leaf extract. Quercetin and its derivates, including rutin and isoquercitrin, are well-known for their antibacterial activity, primarily through disruption of bacterial cell walls and membranes (Ivanov et al., 2022; Nguyen and Bhattacharya, 2022). Their biological activity may be influenced by structural features such as hydroxylation and glycosylation patterns, which can affect physicochemical properties including solubility and interactions with bacterial targets (Nguyen and Bhattacharya, 2022). For instance, previous structure-activity relationship studies suggested that hydroxyl group substitutions at C-5 of the A ring and C-4′ of the B ring, as well as methoxy group substitution at C-3 and C-8 of the A ring are essential for the inhibitory activity of flavonoids (Wu et al., 2013). The contribution of specific structural features remains speculative as individual compounds were not isolated and tested in the present study. Beyond direct antibacterial effects, quercetin has been shown to interfere with quorum sensing, biofilm formation and swarming motility in Gram-negative bacteria such as *P. aeruginosa* (Shastri et al., 2025). In *E. coli*, quercetin has been reported to reduce the expression of genes involved in quorum sensing (luxS), flagellar assembly and motility as well as protease production, thereby reducing bacterial adhesion and inhibiting biofilm development (Li et al., 2024). Notably, quercetin was also used as positive control in the present study and significantly reduced *E. coli* biofilm formation. In addition, kaempferol has also been associated with antimicrobial activity against pathogens including *E. coli* and *S. aureus* (Periferakis et al., 2022). These findings suggest that the observed antimicrobial and anti-virulence effects of *Ribes × nidigrolaria* leaf extracts may, at least in part, be attributed to these flavonoids. However, their specific role requires confirmation by bioassay-guided fractionation and testing of isolated compounds.

Several studies have investigated the antimicrobial activity of plant extracts. However, they focused on a limited number of plant species, individual genera or single target pathogens. Recent examples include studies evaluating selected *Allium* species (Barbu et al., 2023), commercial plant extracts against *S. aureus* biofilms (Barbarossa et al., 2025), or medicinal plants against multidrug-resistant microorganisms (Kebede et al., 2021). In contrast, the present work employed an application-oriented screening approach comprising 200 aqueous plant extracts, originating from non-toxic plant species, evaluated against multiple clinically relevant pathogens. Beyond growth inhibition, anti-virulence activities, including biofilm inhibition and swarming motility reduction, were assessed, enabling the identification of extracts with pathogen-specific activities.

Some limitations should be considered when interpreting the results. Biofilm assays could only be reliably performed for a subset of the investigated pathogens, as *P. aeruginosa* and *E. faecalis* did not lead to robust and reproducible biofilm quantification under the applied assay conditions. Furthermore, swarming motility assays were restricted to *P. aeruginosa* and *S.* Typhimurium, as the remaining pathogens are non-motile. In addition, antibiofilm activity was assessed using a microtiter plate-based assay without additional microscopic, viability-based or molecular analyses. Consequently, changes in biofilm architecture, cell viability or the expression of biofilm-related genes could not be evaluated. Detailed phytochemical characterization by LC-MS was performed only for the promising *Ribes × nidigrolaria* leaf extract, while the remaining extracts were characterized by TPC and antioxidant activity. Further fractionation of active extracts and identification of the underlying bioactive constituents remain the subjects for future studies. Finally, some extracts, such as *Apium graveolens*, showed assay-dependent effects, highlighting the complexity of evaluating crude plant extracts and the need for further validation using complementary methodologies. Nevertheless, the present work successfully identified several locally available aqueous plant extracts with promising antibacterial and anti-virulence activities, highlighting their potential as sustainable alternatives to conventional antibiotics and warranting further investigation of their underlying mechanisms and practical application.

## 5 Conclusion

In conclusion, this study demonstrates a pronounced antibacterial and anti-virulence efficacy of selected plant extracts against clinically relevant pathogens. While strong growth inhibition was mainly observed for *Allium sativum* and *Allium ursinum* extracts, several other aqueous extracts reduced biofilm formation and/or swarming motility at subinhibitory concentrations, highlighting their potential to attenuate pathogenicity. Notably, leaf extracts of *Castanea sativa*, *Rubus laciniatus* and *Ribes × nidigrolaria* showed promising anti-biofilm and anti-swarming activities. To the best of our knowledge, the antimicrobial and anti-virulence activities of *Rubus laciniatus* and *Ribes × nidigrolaria* leaf extracts have not been reported previously. Detailed identification of active constituents remains the subject of future investigations. The selective effect against pathogenic bacteria, while not inhibiting probiotic lactic acid bacteria in the disk diffusion assay, indicates their potential suitability for food and feed applications. Overall, our findings support aqueous plant extracts as promising and application-oriented alternatives to conventional antimicrobials, warranting further investigation into their mechanism of action, efficacy in complex systems and toxicological safety prior to practical use.

## Data Availability

The original contributions presented in this study are included in this article/Supplementary material, further inquiries can be directed to the corresponding author.
