# Supplementary material for: Phenotypic high-content screening identifies plant-derived extracts targeting microbial growth, biofilms and swarming activity
Source: Front Microbiol. 2026 Sep 4;17:1898345. doi: 10.3389/fmicb.2026.1898345 (PMC13585996; doi:10.3389/fmicb.2026.1898345)
Supplement: Supplementary file 1 [file Supplementary_Figures.docx]

Supplementary information

Phenotypic High-content Screening Identifies Plant-Derived Extracts Targeting Microbial Growth, Biofilms and Swarming Activity

Julia Kirchsteiger^1,†^, Verena Preinfalk^1,2^, Gerald Klanert^1,2^, Elisabeth Kohberger^1,2^, Clemens Schwarzinger^3^, Julian Weghuber^1,2*^, Bettina Schwarzinger^1,2,3^ and Alice König^2,†^,

^1^ FFoQSI GmbH – Austrian Competence Centre for Feed and Food Quality, Safety and Innovation, Stelzhamerstraße 23, 4600 Wels, Austria

^2^ Center of Excellence Food Technology and Nutrition, University of Applied Sciences Upper Austria, Stelzhamerstraße 23, 4600 Wels, Austria

^3^ Johannes Kepler University, Institute for Chemical Technology of Organic Materials, Altenberger Straße 69, 4040 Linz, Austria.

^†^These authors contributed equally to this work

*** Correspondence:**
Corresponding Author
julian.weghuber@fh-wels.at

**Supplementary Figure S1.** HPLC-DAD and LC-MS fingerprints of the aqueous *Allium sativum* extract. (a) HPLC-DAD fingerprint. (b) HPLC-PDA chromatogram showing the principal peaks and their relative peak areas. (c) LC-MS total ion chromatogram. The chromatograms are provided as representative fingerprints of the extract used in this study. The retention times (tR) and mass-to-charge ratios (m/z) are summarized in Supplementary Table S10.

**Supplementary Figure S2.** HPLC-DAD and LC-MS fingerprints of the aqueous *Allium ursinum* extract. (a) HPLC-DAD fingerprint. (b) HPLC-PDA chromatogram showing the principal peaks and their relative peak areas. (c) LC-MS total ion chromatogram. The chromatograms are provided as representative fingerprints of the extract used in this study. The retention times (tR) and mass-to-charge ratios (m/z) are summarized in Supplementary Table S10.

**Supplementary Figure S3.** HPLC-DAD and LC-MS fingerprints of the aqueous *Castanea sativa* extract. (a) HPLC-DAD fingerprint. (b) HPLC-PDA chromatogram showing the principal peaks and their relative peak areas. (c) LC-MS total ion chromatogram. The chromatograms are provided as representative fingerprints of the extract used in this study. The retention times (tR) and mass-to-charge ratios (m/z) are summarized in Supplementary Table S10.

**Supplementary Figure S4.** HPLC-DAD and LC-MS fingerprints of the aqueous *Ribes × nidigrolaria* extract. (a) HPLC-DAD fingerprint. (b) HPLC-PDA chromatogram showing the principal peaks and their relative peak areas. (c) LC-MS total ion chromatogram. The chromatograms are provided as representative fingerprints of the extract used in this study. The retention times (tR) and mass-to-charge ratios (m/z) are summarized in Supplementary Table S10.

**Supplementary Figure S5.** HPLC-DAD and LC-MS fingerprints of the aqueous *Rubus laciniatus* extract. (a) HPLC-DAD fingerprint. (b) HPLC-PDA chromatogram showing the principal peaks and their relative peak areas. (c) LC-MS total ion chromatogram. The chromatograms are provided as representative fingerprints of the extract used in this study. The retention times (tR) and mass-to-charge ratios (m/z) are summarized in Supplementary Table S10.
